# Distribution of Anophelinae (Diptera: Culicidae) and challenges for malaria elimination in Brazil

**DOI:** 10.1590/0074-02760240247

**Published:** 2025-02-24

**Authors:** Maria Anice Mureb Sallum, Thiago Salomão de Azevedo, Jan Evelyn Conn, Ricardo Lourenço-de-Oliveira

**Affiliations:** 1 Universidade de São Paulo, Faculdade de Saúde Pública, Departamento de Epidemiologia, São Paulo, SP, Brasil; 2 Secretaria da Saúde de Santa Bárbara d’Oeste, Santa Bárbara d’Oeste, SP, Brasil; 3 Wadsworth Center, New York State Department of Health, Albany, NY, USA; 4 Fundação Oswaldo Cruz-Fiocruz, Instituto Oswaldo Cruz, Laboratório de Mosquitos Transmissores de Hematozoários, Rio de Janeiro, RJ, Brasil

**Keywords:** Anophelinae, malaria, vector distribution, land use change, Plasmodium, Brazil

## Abstract

In 1909, Arthur Neiva published an article titled “*Contribuição para os estudos dos dipteros. Observação sobre a biolojia e sistematica das anofelinas brasileiras e suas relações com o impaludismo*”, highlighting the biology, ecology, and distribution of Anophelinae mosquitoes and the need for more taxonomic studies in Brazil. This came 11 years after Ronald Ross and Grassi demonstrated mosquito roles in transmitting *Plasmodium* to birds and humans. Despite considerable advances in the understanding of Anophelinae species, knowledge remains insufficient given the complexity of Brazil’s ecosystems, the intensified anthropogenic environmental changes since the mid-20th century, and the persistent public health challenges posed by malaria. This perspective article presents the distribution of *Plasmodium* vectors and potential vector species in Brazil using climate variables and a maximum entropy model. Geographical distribution maps of Anophelinae species, including putative species, are provided. The article also discusses the current knowledge of vector species distribution in relation to Brazil’s malaria elimination plan, along with the ecological and anthropogenic factors influencing vector distribution.

In 1909, Arthur Neiva published an article in Portuguese and German titled “*Contribuição para os estudos dos dipteros. Observação sobre a biolojia e sistematica das anofelinas brasileiras e suas relações com o impaludismo*”.^1^ This followed the groundbreaking 1898 discovery by Sir Ronald Ross, who demonstrated the role of mosquitoes in bird malaria transmission, leading to significant research on the systematics, distribution, and bionomics of Anophelinae Grassi mosquitoes globally. At that time, the genera with representatives in Brazil were ― *Myzomyia* Blanchard (currently synonymous with *Cellia* Theobald), *Cyclolepteron* Theobald (currently synonymous with *Anopheles* Meigen), *Stethomyia* Theobald, *Myzorynchella* Theobald (currently synonymous with *Nyssorhynchus* Blanchard), *Arribalzagia* Blanchard (currently synonymous with *Anopheles*), *Cellia*, *Chagasia* Cruz and *Manguinhosia* Cruz (currently synonymous with *Nyssorhynchus*).

In his article, Neiva detailed the known geographic distribution of Brazilian mosquito species (Fig. 1) and discussed various aspects of their biology, ecology, and blood-feeding behaviors. Upon reviewing the article, two aspects stand out as particularly striking. First, the meticulous detail with which the knowledge of these mosquitoes was presented. Second, when addressing the challenges of locating larval habitats, Neiva emphasized the scarcity of studies on the biology of *Anopheles* species in Brazil - a gap that, 115 years later, remains largely unaddressed. An important early study worth highlighting is the field entomological investigations conducted from 1939 to 1944 by Deane, Causey, and Deane. Using various sampling methods, their research provided invaluable data on the biology, ecology, and distribution of 36 anopheline species identified in the northeastern and Amazon regions of Brazil, covering over 4.5 million square kilometers.^2^ Despite advances in scientific knowledge, research has predominantly focused on species of public health significance, leaving gaps in the broader understanding of Anophelinae taxonomy, biology, and ecology in Brazil.

**Fig. 1:**
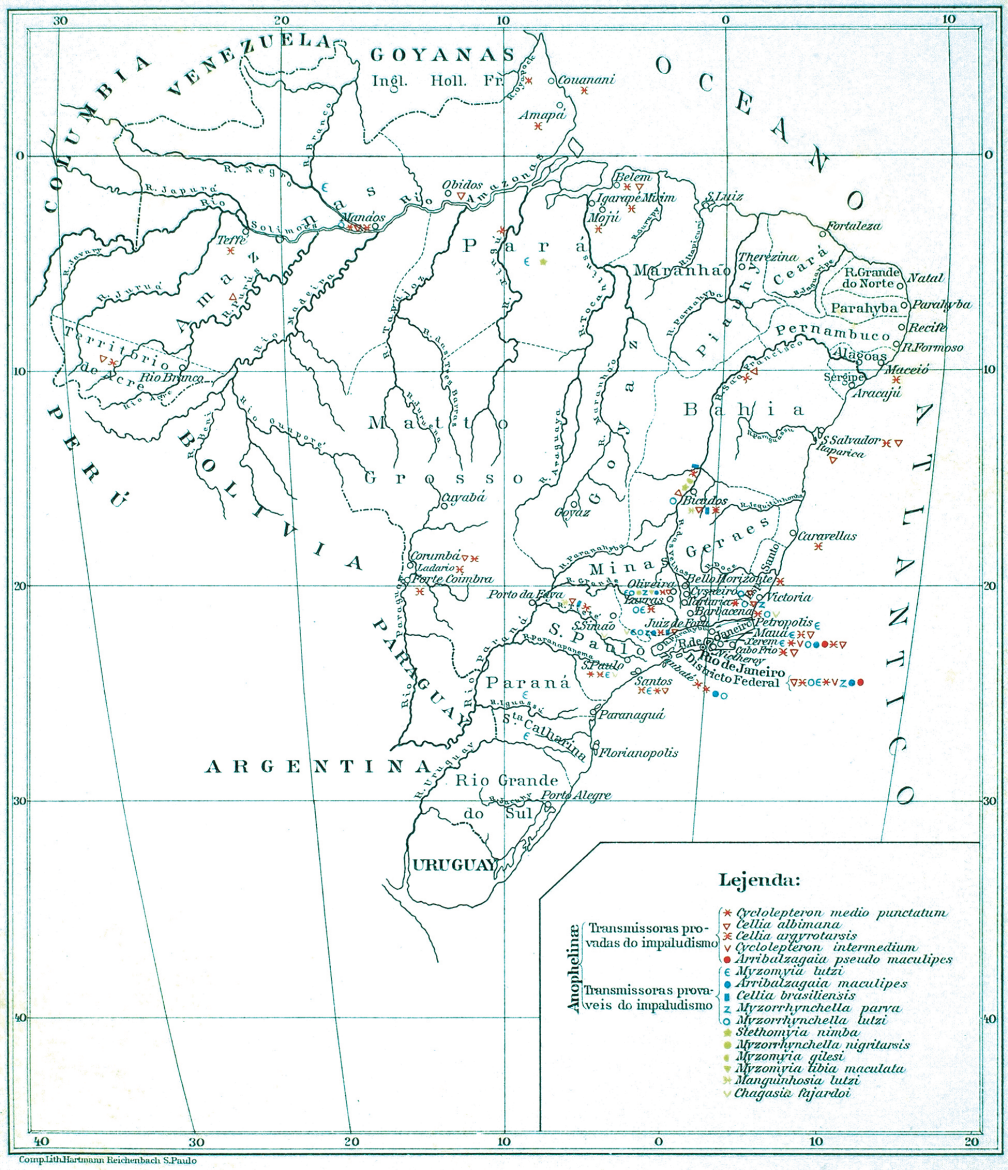
Neiva’s 1909 map detailing the known geographic distribution of Brazilian mosquito species.

In honor of the 115th anniversary of Memórias do Instituto Oswaldo Cruz, and with the respect Professor Neiva’s pioneering work deserves, this perspective article focuses on the potential geographic distribution of *Nyssorhynchus darlingi* (Root) and *Kerteszia cruzii* (Dyar & Knab) in Brazil. Additionally, distribution maps for other anopheline species are provided to showcase the diversity of species found in the country. The distribution analyses are primarily based on recent data from field collections, from published literature records, and specimens from the Entomological Collection of the Faculdade de Saúde Pública at the Universidade de São Paulo (CER-FSP), Brazil. The study also reflects on how gaps in knowledge of species distributions complicate malaria vector surveillance efforts, particularly in the context of Brazil’s malaria elimination initiatives.

Malaria as a public health concern

Malaria remains a significant public health concern in Brazil, particularly in the Amazon region, where over 99% of cases are reported.^3^ Despite considerable efforts to control and reduce the disease incidence, malaria continues to affect several thousands of people annually, leading to loss of productivity, poverty, and jeopardizing the local health services in endemic countries.^4^ ^,^ ^5^ ^,^ ^6^ Malaria impact is most pronounced in indigenous people, rural communities and remote illegal mining, where lack or poor access to fast diagnostic, antimalarial drugs, and molecular tools to monitor transmission, challenge the effective control and elimination of the disease.^7^ ^,^ ^8^ In the Amazon, human migration patterns, especially the interstate travel network of infected people, can have an impact on the Brazil goals of malaria elimination by 2035.^3^ Similarly, the risk of malaria parasite propagation is not negligible by nonimmune travelers within the Amazon and from the Amazon to outside regions where malaria was either eliminated or transmission is low.^9^

Malaria has been a significant public health challenge in Brazil for centuries, with its history deeply intertwined with the country’s socio-economic development and environmental changes.^10^ In the early 20th century, malaria was widespread throughout much of Brazil, particularly in the Parana River, Amazon River basin, and on the Atlantic coast, where the highest transmission rates occurred.^11^ The invasion of *Anopheles* (*Cellia*) *gambiae* Giles, later identified as *Anopheles* (*Cellia*) *arabiensis* Patton^12^ in the northeast state of Rio Grande do Norte and its spread to Ceará State was a major challenge to malaria control in the 1930s.^13^ ^,^ ^14^ In the early 1940s, after approximately 14,000 deaths and 150,000 cases in the northeastern states, *An. arabiensis* was eliminated from Brazil.^15^ ^,^ ^16^ Efforts to control malaria intensified in the mid-20th century, leading to notable declines in cases because of the widespread use of insecticide improved surveillance, and public health campaigns.^11^ The resurgence of cases in the 1980s highlighted the limitations of these strategies, particularly in regions undergoing rapid deforestation, infrastructure development, and population migration, primarily associated with the expansion of agrobusiness and exploitation of basic commodities for global trade.^10^ ^,^ ^17^

The Amazon region remains the epicenter of malaria transmission in Brazil, accounting for most cases nationwide. Within this region, continuous transmission zones are typically concentrated in remote, forested areas, particularly along rivers, where *Ny. darlingi*, the dominant mosquito vector, thrives.^18^ These areas often coincide with regions experiencing rapid deforestation, illegal mining, agricultural expansion, and infrastructure development, all of which create favorable conditions for mosquito habitats. Migration and mobility of human populations further exacerbate transmission, as workers and settlers in newly developed areas can spread *Plasmodium* spp. to regions with limited healthcare access.^3^ States such as Amazonas, Acre, and Pará consistently report the highest malaria incidences, with indigenous communities and rural populations being the most vulnerable.^19^ Despite ongoing control efforts, such as the distribution of insecticide-treated nets and the expansion of diagnostic and treatment services, the complex socio-environmental dynamics of the Amazon region pose significant challenges to malaria elimination.^19^

Taxonomy of Anophelinae

The subfamily Anophelinae Grassi typically includes the genera *Anopheles* Meigen, *Bironella* Theobald, and *Chagasia* Cruz.^20^ The species of the genus *Anopheles* were classified into the subgenera *Anopheles* Meigen, *Baimaia* Harbach, Rattanarithikul, Harrisson, *Cellia* Theobald, *Christya* Theobald, *Kerteszia* Theobald, *Lophopodomyia* Antunes, *Nyssorhynchus* Blanchard, and *Stethomyia* Theobald. However, Foster et al.^21^ elevated *Kerteszia*, *Lophopodomyia*, *Nyssorhynchus* and *Stethomyia* to the genus level, resulting in a total of seven genera in Anophelinae. The genus *Anopheles*, in turn, now includes the subgenera *Anopheles*, *Baimaia*, *Cellia*, and *Christya*. The species of public health importance belong to the genus *Anopheles*, subgenera *Anopheles* (cosmopolitan) and *Cellia* (Old World), genus *Kerteszia* (Neotropics), and genus *Nyssorhynchus* (Neotropics and southern Nearctic).

Mosquitoes of the subfamily Anophelinae are found worldwide, except in few locations and islands where the ecological factors and temperature are inadequate for mosquitoes.^22^ Females are responsible for transmitting four species and two subspecies of the protozoan genus *Plasmodium* Marchiafava & Celli that cause human malaria.^23^ Approximately 70 out of approximately 500 valid species are dominant vectors of *Plasmodium*.^24^ In addition, certain species play a role in transmitting arboviruses, such as O’nyong-nyong,^25^ ^,^ ^26^ Guaroa,^27^ and might be efficient vectors of Mayaro and Sindbis viruses based on results of recent laboratory experiments.^28^ In the Americas, nine species or species of complexes are dominant vectors of *Plasmodium* spp.: *Nyssorhynchus albimanus* (Wiedemann), *Nyssorhynchus albitarsis* complex, *Nyssorhynchus aquasalis* (Curry), *Ny. darlingi* (Root), *An*. (*Anopheles*) *freeborni* Aitken, *Nyssorhynchus marajoara* (Galvão & Damasceno), *Nyssorhynchus nuneztovari* complex, *An*. (*Anopheles*) *pseudopunctipennis* complex, and *An*. (*Anopheles*) *quadrimaculatus* Say.^24^ ^,^ ^29^

In Brazil, *Ny. darlingi* is the dominant vector in areas across the Amazon River basin, *Ny. aquasalis* is an effective vector in areas on the Atlantic coast, while *Ke. cruzii* (Dyar & Knab), *Ke. bellatrix* (Dyar & Knab), and *Kerteszia homunculus* Komp are vectors in the Atlantic Forest biome.^30^ In addition, there are other species that were found naturally infected and may be local vectors of *Plasmodium vivax* (Grassi & Feletti), and/or *Plasmodium falciparum* (Welch): *Anopheles* (*Anopheles*) *peryassui* Dyar & Knab, *Nyssorhynchus benarrochi* B, *Nyssorhynchus tadei* (Saraiva & Scarpassa), *Nyssorhynchus oswaldoi* A, *Nyssorhynchus rangeli* (Gabaldón, Cova Garcia & Lopez), *Nyssorhynchus triannulatus* (Neiva & Pinto), among other species listed in Supplementary data (Table I).

Drivers of Anophelinae distribution and malaria transmission

The geographic distribution of Anophelinae species is influenced by environmental factors such as precipitation, temperature, altitude, availability of larval habitats, relief and hydrology.^31^ However, changes in the natural environment and land use can create micro and macroenvironmental conditions adequate for anopheline species proliferation and dispersion. *Ny. darlingi* is found in areas of extensive forest cover and along river networks from northern to southern Brazil, reaching its known south limit at the Foz do Iguaçu, Paraná State. In well preserved forest in Yanomami lands, in the Brazilian Amazon, the species is found in lakes associated with river floodplains, and old river paths with U-shaped form (oxbow lakes) that were formed by river isolation because of sedimentation or erosion.^32^ ^,^ ^33^ In landscapes impacted by anthropic modifications in the natural environment, *Ny. darlingi* aquatic stages are found in lagoons, streams, streams combined with lagoons, streams combined with dams, and fishponds in the Brazilian Amazon.^34^ ^,^ ^35^ ^,^ ^36^ ^,^ ^37^

*Kerteszia cruzii*, *Ke. bellatrix*, and *Ke. homunculus* are sylvatic mosquitoes and vectors of malaria parasites in the Atlantic Forest biome in southeastern Brazil. These species occur in areas where bromeliad plants are abundant because they depend on these plant phytotelmas for egg laying and development of aquatic stages.^38^ Particularly, *Ke*. *cruzii* is a primary vector in areas where the anthropogenic changes are altering mosquito dynamics and malaria risk, especially in forested areas in São Paulo, Rio de Janeiro, Espírito Santo, Santa Catarina, and northeastern Rio Grande do Sul.^39^ ^,^ ^40^ ^,^ ^41^ The first documented association of *Ke*. *cruzii* as a natural vector of simian *Plasmodium simium* Marchiafava & Celli and *Plasmodium brasilianum* (Gonder & Von Berenberg-Gossler) in the Atlantic Forest was reported by Deane et al.^42^ However, both *Plasmodium* spp. can infect and cause infection and malaria in humans when they encroach on forest environments.^43^ The first report of *P. simium* malaria in humans was registered by Deane et al.^44^ in the forest reservation of Horto Florestal in Serra da Cantareira, north São Paulo municipality, São Paulo State. During two-year collections in the canopy and on the ground level, Deane and colleagues collected 30 mosquito species, among them eight anopheline species, including *Ke. cruzii* that was the most abundant on both level ground and in the canopy, and thus was considered a potential vector. Further studies in other states across the Atlantic Forest, Cerrado, and Amazon were crucial to bring to light new knowledge about the sylvatic reservoirs of the simian *Plasmodium* and mosquito vectors.^45^ ^-^ ^52^ Under current and future climate scenarios, both *P. vivax* and *P. falciparum* may experience shorter extrinsic incubation periods, while their vector, *Ke. cruzii*, may increase survival rates because of rising temperatures. This could lead to the expansion of high-risk malaria areas, particularly in the southern Atlantic Forest.^53^

In northeast Brazil, *Ny. aquasalis* plays a role in transmitting malaria parasites in coastal communities, as well as in other coastal areas in South and Central America and Caribbean Island.^54^ Females lay their eggs in a mixture of fresh and saltwater typically found in coastal lagoons, estuaries, and mangroves. In the mangrove ecosystem, *Ny. aquasalis* larval and pupal stages can be found in stagnant or slow-moving waters in mangrove swamps, in full sun or partially shaded with mangrove vegetation, grasses and algae.^55^ Tidal pools with varying salinity levels are also used as larval and pupal stage habitats. *Ny. aquasalis* may also breed in human-made habitats such as fishponds, salt marshes, and drainage canals near coastal areas.^55^ The adaptability of the species to a range of salinity levels allows it to occupy a unique reproductive niche not occupied by other anopheline species in coastal regions of northeastern Brazil and other parts of South America.^38^ ^,^ ^55^

Anthropogenic changes in land use, particularly deforestation and agricultural expansion, have a profound impact on the distribution and dynamics of mosquito communities.^56^ Deforestation, habitat fragmentation, and the reduction of forest cover can drastically affect mosquito biodiversity, leading to the decline of some species while others, resilient to altered environments, become dominant.^57^ For example, in the northeastern Brazilian Amazon, *Ny. darlingi*, traditionally the primary malaria vector, was replaced by *Ny. marajoara* because of changes in land use, with *Ny. marajoara* emerging as the dominant vector species.^58^ *Ny. marajoara* is classified in the Albitarsis Complex that encompasses five valid species and five putative species to be formally named. Despite the difficulties in the identification of the species based solely on external characteristics, molecular analyses have confirmed the identification of distinct species within this complex.^59^ Members of this group are typically associated with aquatic habitats where their larvae develop, such as irrigated rice fields, dam, fishponds, lakes, ponds, streams in full sun, and partially shaded and shaded water.^36^ ^,^ ^60^ In rural settlements in the Brazilian Amazon, *Ny. darlingi* benefits from deforestation, becoming more prevalent in areas with higher forest cover and lower border density.^61^ These findings suggest that deforestation and forest fragmentation create environmental conditions that favor shifts in mosquito community structure, with significant changes in species dominance and distribution that may increase human exposure to vectors of malaria parasites, heightening the risk of vector-borne disease worldwide.^62^

*Nyssorhynchus darlingi* is, to some extent, resilient to the ecological conditions in human-modified environments.^61^ As urbanization expands and natural habitats are modified, some anopheline mosquitoes are adjusting to new ecological settings by exploiting new habitats, such as human-made water bodies, drains, lagoons, lakes, and ponds in fish farms.^34^ ^,^ ^36^ ^,^ ^37^ *Ny. darlingi* becomes increasingly abundant and dominant in peridomestic environments of human settlements because of anthropogenic factors such as deforestation, alterations in water ecosystems, the creation of standing water bodies, and other human-made structures.^61^ The reduction of forest cover and the rise in edge density associated with deforestation result in decreased mosquito biodiversity, creating favorable conditions for the proliferation of *Ny. darling*.^61^ One reason for the increased abundance of *Ny. darlingi* is the ecological advantage of its adaptation of the use of small dams in natural water bodies in forest fringes as larval habitats.^63^ The link between deforestation and *Ny. darlingi* was also found in areas with varying landscape composition in the Peruvian Amazon forest. Larvae of *Ny. darlingi* were found in water systems in areas with an average of 24.1% forest cover, contrasting with areas without the species that had 41.0% forest cover. Seasonality, presence of algae, water body size, presence of human populations, and the amount of forest and secondary growth were found to be significant for the presence of larval habitats and *Ny. darlingi* presence.^64^ In another study conducted in the Brazilian Amazon, spatial clustering of *Ny. darlingi* larvae was observed in areas where obstructions to river flow created slow-moving or stagnant pools of water. These conditions, combined with reduced sunlight exposure, favor *Ny. darlingi* larval habitats.^35^ ^,^ ^65^

Understanding the geographical distribution of Anophelinae species that are well-known and those that are potential vectors of malaria is crucial for the ongoing efforts toward malaria elimination.^66^ ^,^ ^67^ Each species has a unique ecological niche, behavior, blood feeding behavior, and vectorial capacity, meaning that the risk of a susceptible person acquiring malaria varies significantly across different landscapes.^68^ Mapping vector species distribution may help identify areas of higher transmission risk, thus enabling targeted interventions, such as environmental management.^69^ Also, geospatial distribution modeling together with parasite serology can be employed to help to identify foci of residual malaria transmission.^70^

At the same time as malaria elimination advances, understanding the distribution of malaria vectors becomes critical for sustaining the success of interventions.^71^ In areas where malaria has been largely eliminated but transmission remains uneven, the disease epidemiology becomes more complex.^71^ ^,^ ^72^ Therefore, it is essential to establish an active vector surveillance program capable of detecting areas with residual transmission pockets, where malaria vector populations persist at low density.^73^ In addition, knowledge of Anophelinae distribution potential allows for early detection of shifts in vector populations because of changes in the environments, land use, urbanization, and climate.^74^ In regions where malaria transmission has been reduced or eliminated, monitoring the presence and expansion of mosquito vectors is critical to sustaining elimination achievements and preventing the resurgence of malaria.^73^ In addition, to achieve the long-term goal of malaria eradication it will be necessary to have greater funding, innovative solutions, and global cooperation.^75^

Climate change can drive shifts in the geographic distribution of mosquito vectors of *Plasmodium* and alter malaria-endemic areas. These changes will depend on the environment suitability for malaria transmission, thermal tolerance limits of both mosquito vectors and *Plasmodium* parasites, the availability of suitable habitats for aquatic life stages, and hydrological processes.^31^ ^,^ ^76^ Additionally, climate changes can cause alterations in blood-feeding behavior, availability of vertebrate hosts, mosquito life cycle duration, female mosquito longevity, and the duration of *Plasmodium* extrinsic incubation period can influence malaria dynamics and seasonality.^77^ ^,^ ^78^ Variation in temperature affects the development of *Ny. darlingi* in Brazil. Comparing three populations from Brazil, Amazon, Cerrado, and Atlantic Forest, it was found that higher temperatures accelerated larval development, but also shortened adult lifespan and overall longevity, while reducing body size at 28ºC compared to 20ºC. However, *Ny. darlingi* population from the Atlantic Forest exhibited faster development at warmer temperatures, while maintained a larger body size compared to other populations studied and showed no reduction in longevity.^79^ These factors together suggest that the vectorial capacity of *Ny. darlingi* from the Atlantic Forest population may increase under warmer conditions. Moreover, higher temperatures can expand the geographical range of Anophelinae mosquitoes into previously cooler areas, including highland regions, raising malaria risk in areas that were previously non-endemic.^80^ ^,^ ^81^ ^,^ ^82^ In summary, climate change can exacerbate malaria transmission in some areas, while in others, it can reduce the disease.

Ecological distribution of Anophelinae in Brazil

Since Professor Neiva’s inspiring article in 1909, numerous anopheline species have been described and named. Over time, changes in nomenclature have been proposed, reducing the number of Anophelinae genera from 20 to three, and increasing to seven globally in 2017.^21^ In Brazil, Neiva recorded eight genera in 1909, which later decreased to two and then increased to six by 2017. To align with the nomenclature proposed by Foster et al.,^21^ corrections to species names regarding gender and authority citations are necessary, as outlined in Tables I and II.

**TABLE I:** Valid species of the genus *Anopheles* subgenus *Anopheles*, and the genera *Kerteszia*, *Lophopodomyia*, and *Stethomyia* found in South America, grouped by subgenera and series, with updates to authority citations and corrections to gender classification. Species marked with an asterisk have been recorded in Brazil

Genus/Series	Species, author, date
*Anopheles* Meigen, 1818 Series Arribalzagia	*anchietai* Corrêa & Ramalho, 1968^*^
	*annulipalpis* Lynch Arribálzaga, 1878
	*apicimacula* Dyar & Knab, 1906^*^
	*bustamantei* Galvão, 1955^*^
	*calderoni* Wilkerson, 1991
	*costai* da Fonseca & da Silva Ramos, 1940^*^
	*evandroi* da Costa Lima, 1937^*^
	*fluminensis* Root, 1927^*^
	*forattinii* Wilkerson & Sallum, 1999^*^
	*guarao* Anduze & Capdevielle, 1949^*^
	*maculipes* (Theobald, 1903)^*^
	*malefactor* Dyar & Knab, 1907
	*mattogrossensis* Lutz & Neiva, 1911^*^
	*medialis* Harbach, 2018 (new name for *Anopheles intermedius* Chagas, 1908)^*^
	*mediopunctatus* (Lutz, 1903)^*^
	*minor* da Costa Lima, 1929^*^
	*neomaculipalpus* Curry, 1931^*^
	*peryassui* Dyar & Knab, 1908^*^
	*pseudomaculipes* (Chagas in Peryassú, 1908)^*^
	*punctimacula* Dyar & Knab, 1906^*^
	*rachoui* Galvão, 1952^*^
	*shannoni* Davis, 1931^*^
	*vestitipennis* Dyar & Knab, 1906
Series Anopheles	*eiseni eiseni* Coquillett, 1902
	*eiseni geometricus* Corrêa, 1944^*^
	*pseudopunctipennis levicastilloi* Levi Castillo, 1944
	*pseudopunctipennis neghmei* Mann, 1950
	*pseudopunctipennis noei* Mann, 1950
	*pseudopunctipennis patersoni* Alvarado & Heredia, 1947
	*pseudopunctipennis pseudopunctipennis* Theobald, 1901
	*pseudopunctipennis rivadeneirai* Levi Castillo, 1945
	*tibiamaculatus* (Neiva, 1906)^*^
*Kerteszia* Theobald, 1905	*auyantepuiensis* (Harbach & Navarro, 1996)
	*bambusicola* (Komp, 1937)^*^
	*bellatrix* (Dyar & Knab, 1906)^*^
	*boliviensis* Theobald, 1905
	*cruzii* (Dyar & Knab, 1908)^*^
	*gonzalezrinconesi* (Cova-García, Pulido F. & Escalante de Ugueto, 1977)
	*homunculus* (Komp, 1937)^*^
	*laneana* (Corrêa & Cerqueira, 1944)^*^
	*lepidota* (Zavortink, 1973)^*^
	*neivai* (Howard, Dyar & Knab, 1913)^*^
	*pholidota* (Zavortink, 1973)
	*rollai* (Cova-García, Pulido F. & Escalante de Ugueto, 1977)
*Lophopodomyia* Antunes, 1937	*gilesi* (Neiva in Peryassú, 1908)^*^
	*gomezdelatorrei* (Leví-Castillo, 1955)
	*oiketorakras* (Osorno-Mesa, 1947)
	*pseudotibiamaculata* (Galvão & Barretto, 1941)^*^
	*squamifemur* (Antunes, 1937)^*^
	*vargasi* Gabaldón, (Cova García & López, 1941)
*Stethomyia* Theobald, 1902	*acanthotoryna* (Komp, 1937)
	*canorii* (Floch & Abonnenc, 1945)
	*kompi* (Edwards, 1930)^*^
	*nimbus* Theobald, 1902^*^
	*thomasi* (Shannon, 1933)^*^

**TABLE II:** Valid species of the genus *Nyssorhynchus* (subfamily Anophelinae) found in South America, grouped by series and informal groups, with updates to authority citations and corrections to gender classification. Species marked with an asterisk have been recorded in Brazil

Series	Group	Subgroup	Complex	Species, author, date
Albimanus				*albimanus* (Wiedemann, 1820)
Oswaldoi	Oswaldoi	Oswaldoi		*aquasalis* (Curry, 1932)^*^
				*evansae* (Brèthes, 1926)^*^
				*galvaoi* (Causey, Deane & Deane, 1943)^*^
				*ininii* (Senevet & Abonnenc, 1938)^*^
				*oswaldoi* (Peryassú, 1922) (*s.s.*)^*^
				*rangeli* (Gabaldon, Cova Garcia & López, 1940)^*^
				*sanctielii* (Senevet & Abonnenc, 1938)
				*trinkae* (Faran, 1979)
			Konderi	*konderi* (Galvão & Damasceno, 1942) (s.s.)^*^
				*tadei* (Saraiva & Scarpassa, 2021)^*^
			Nuneztovari	*dunhami* (Causey, 1945)^*^
				*goeldii* (Rozeboom & Gabaldon, 1941)^*^
				*jamariensis* Sant’Ana & Sallum, 2024^*^
				*nuneztovari* (Gabaldon, 1940) (*s.s.*)^*^
		Strodei	Arthuri	*albertoi* (Unti, 1941)^*^
				*arthuri* (Unti, 1941) (s.s.)^*^
				*ibiapabaensis* Sant’Ana & Sallum, 2024^*^
				*rondoni* (Neiva & Pinto, 1922)^*^
				*rondoniensis* Sant’Ana & Sallum, 2024^*^
				*striatus* (Sant’Ana & Sallum, 2016)^*^
				*strodei* (Root, 1926)^*^
				*untii* Sant’Ana & Sallum, 2024^*^
			Benarrochi	*benarrochi* (Gabaldon, Cova Garcia & López, 1941) (s.s.)
		Triannulatus		*halophylus* (Silva-do-Nascimento & Lourenço-de-Oliveira, 2002)^*^
				*triannulatus* (Neiva & Pinto, 1922) (s.s.)^*^
Albitarsis	Albitarsis		Albitarsis	*albitarsis* (Lynch Arribálzaga, 1878) (s.s.)^*^
				*deaneorum* (Rosa-Freitas, 1989)^*^
				*janconnae* (Wilkerson & Sallum, 2009)^*^
				*marajoara* (Galvão & Damasceno, 1942)^*^
				*oryzalimnetes* (Wilkerson & Motoki, 2009)^*^
	Braziliensis			*braziliensis* (Chagas, 1907)^*^
Argyritarsis	Argyritarsis			*argyritarsis* (Robineau-Desvoidy, 1827)^*^
				*sawyeri* (Causey, Deane, Deane & Sampaio, 1943)^*^
	Darlingi			*darlingi* (Root, 1926)^*^
	Lanei			*lanei* (Galvão & Franco do Amaral, 1938)^*^
	Pictipennis			*atacamensis* (González & Sallum, 2010)
				*pictipennis* (Philippi, 1865)
Myzorhynchella				*antunesi* (Galvão & Franco do Amaral, 1940)^*^
				*guarani* (Shannon, 1928)^*^
				*lutzii* (Cruz, 1901) (s.s.)^*^
				*nigritarsis* (Chagas, 1907)^*^
				*parvus* (Chagas, 1907)^*^
				*pristinus* (Nagaki & Sallum, 2010)^*^

Currently, approximately 103 species of Anophelinae have been documented in Brazil, including 73 formally described species and 30 putative species identified through *COI* barcoding and supported by morphological evidence [Supplementary data (Table I)]. These putative species, while recognized through genetic and morphological divergence, remain to be formally described [Supplementary data (Table I)]. The species recorded in Brazil belong to the genera *Anopheles*, *Chagasia*, *Kerteszia*, *Lophopodomyia*, *Nyssorhynchus*, and *Stethomyia*. The genus *Kerteszia* includes seven formally named species and two additional putative species identified through *COI* barcoding of the mitochondrial genome.^83^ The genus *Nyssorhynchus* comprises 36 formally named species and at least 19 yet-to-be-described species, such as *An*. *albitarsis* F-J,^84^ *An. oswaldoi* A, B, *An. benarrochi* B, *An. konderi* A, C, D, *An. triannulatus* C,^85^ among others [Supplementary data (Table I)]. Additionally, species of the *Myzorhynchella* subgenus require further study, as field collections across various Brazilian localities have revealed evidence supporting the existence of several new species, based on morphological characteristics of males, females, fourth-instar larvae, and *COI* barcode sequences.^86^ ^,^ ^87^ The genus *Anopheles* includes 22 species, and 10 potential new species identified via *COI* barcoding,^88^ ^,^ ^89^ some of which have proved to be experimentally competent to transmit malaria parasites^90^ ^,^ ^91^ and/or found naturally infected. The least studied genera, *Stethomyia*, *Lophopodomyia*, and *Chagasia*, each have three species recorded in Brazil. These exclusively Neotropical genera are likely under-sampled and poorly understood due to challenges in collecting immature stages and adults, as well as their limited or unknown public health relevance as vectors of human *Plasmodium*.

The geographic distribution of *Ke. cruzii* is primarily restricted to the Atlantic Forest biome (Fig. 2A). However, the coastline extending from northern Santa Catarina to northern São Paulo states represents the areas with the highest climatic suitability for this species [Fig. 2C, Supplementary data (Table II)]. *Ny. darlingi* has a wide distribution across Brazil and is found in all major biomes except the Pampas (Fig. 2B). Nevertheless, certain regions of Brazil exhibit low habitat suitability for *Ny*. *darlingi* [Fig. 2D, Supplementary data (Table II)]. These areas include southern Brazil, particularly in Rio Grande do Sul, and transitional zones between the Pampas and Atlantic Forest biomes in Santa Catarina and southern Paraná states. Additionally, low suitability is noted in the Mantiqueira Mountain Range, located at the intersection of São Paulo, Minas Gerais, and Rio de Janeiro states. Further, *Ny*. *darlingi* shows low probability of occurrence in the Zona da Mata region, the tablelands of Bahia State, and the plateau regions of the western Amazon. On the other hand, areas with high susceptibility for *Ny*. *darlingi* occurrence include the Acaraú River valley near the Paulo Sarasate dam, at the foothills of the Ibiapaba Mountain Range in Ceará State. Other regions of high suitability include the Sertanejos Residual Plateaus of Seridó, Serra de Santana, and Vale do Açu, situated on the border between Paraíba and Rio Grande do Norte states (Fig. 2D).

**Fig. 2:**
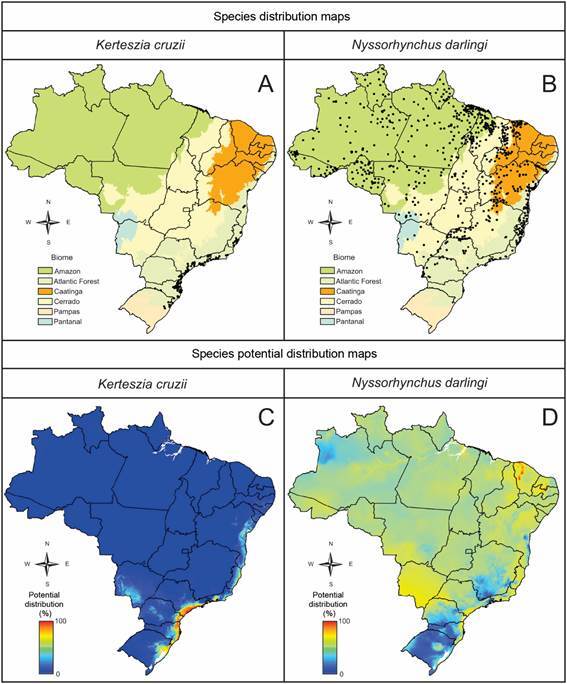
the probability of presence (ranging from 0 to 1) for *Nyssorhynchus darlingi* and *Kerteszia cruzii* was modeled based on the geographical areas with optimal climate conditions, as identified using climate variables from the WorldClim Global Climate Data. The analysis was conducted at a spatial resolution of 30 arc seconds (approximately 1 km), using a maximum entropy distribution model.

Mapping potential distribution of Anophelinae vector species and diversity

Currently, approximately 103 species of Anophelinae have been documented in Brazil, including 30 putative species that have yet to receive formal naming [Supplementary data (Table I)]. Among these, 30 species and one species complex have been identified as dominant, local, or potential vectors. These species have either been found naturally infected, demonstrated vector competence in laboratory conditions, are known vectors in bordering countries, or have been detected infected in peridomestic environments, and forested areas with endemic transmission [Figs 2,3, Supplementary data (Table I)].

Results of climate variables selection and principal components analyses (PCA) are in the Supplementary data (Tables II-III). The MaxEnt modeling analysis incorporated the four most representative eigenvectors of climate variables relevant to the vector species under study [Supplementary data (Table III)]. In addition, the MaxEnt analyses indicate that the key climatic factors influencing the distribution of *Ny. darlingi* and *Ke. cruzii* are the minimum temperature of the coldest month, temperature annual range, and precipitation-related variables, with some variation in the specific importance of these factors between species. While precipitation seasonality and the minimum temperature of the coldest month are particularly important for *Ny. darlingi*, isothermality and the precipitation of the driest month are the most influential factors for *Ke. cruzii*. MaxEnt modelling produced varying predictive accuracies, with an AUC score of 0.70 for *Ny. darlingi* (moderate accuracy) and 0.98 for *Ke. cruzii* (high accuracy) [Supplementary data (Table IV)].

The potential geographic distribution of anopheline species with some role in malaria transmission dynamics, influenced by climatic factors, is highly heterogeneous. Most species are predominantly confined to the Amazon region, characterized by its warm and humid climate (Fig. 3). However, certain species have broader distributions across Brazilian biomes. These more adaptable, generalist species include *An. medialis*, *An. peryassui*, *Ke. homunculus*, *Ny. darlingi*, *Ny. rangeli*, *Ny*. *rondoniensis*, and *Ny. triannulatus*. Species such as *Ke. bellatrix* and *Ke. cruzii* thrives in the Atlantic coastal climate, especially in the northeast, where conditions are humid and tropical. The Atlantic Forest includes altitude tropical climates in southeastern Brazil, characterized by consistently high temperatures and humidity, with well-distributed rainfall throughout the year. Distinct from other vectors of *Plasmodium*, *Ny. strodei* is suited to both tropical and temperate climates. It occupies areas with mild average temperatures and dry winters, with its geographic distribution extending to the southernmost parts of Brazil.

**Fig. 3:**
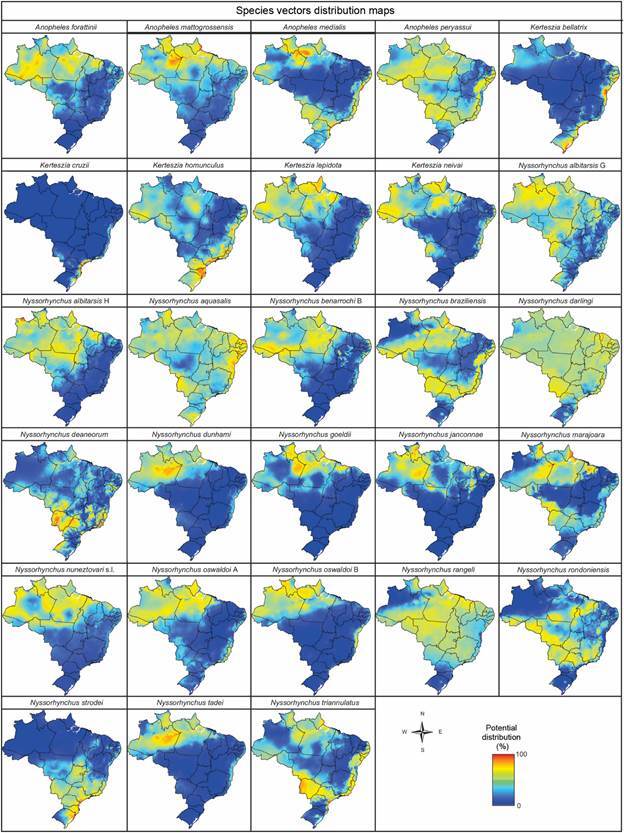
the probability of presence (ranging from 0 to 1) for 28 Anophelinae species or putative species with some role in malaria transmission dynamics was modeled based on the geographical areas with optimal climate conditions, as identified using climate variables from the WorldClim Global Climate Data. The analysis was conducted at a spatial resolution of 30 arc seconds (approximately 1 km), using a maximum entropy distribution model.

The Shannon-Wiener diversity map shows that regions with the highest diversity of anopheline vectors are largely concentrated in the Amazon (Fig. 4). The Brazilian Atlantic Coast, particularly rainforest areas, also exhibits high vector diversity, notably in the northeastern Zona da Mata. Other areas of significant diversity include Monte Pascoal National Park in Bahia and northern Espírito Santo. In the Southeast, regions with considerable diversity include Fluminense, Costa do Sol, and Guanabara Bay. Additionally, the border area between São Paulo and Paraná, which encompasses the São Paulo State parks of Lagamar de Cananéia and Ilha do Cardoso as well as Superagüi National Park in Paraná, hosts high anopheline diversity. The Mato Grosso and Rondônia ecotones between the Pantanal and Amazon regions, in western Mato Grosso and Rondônia, are areas of notable diversity where *Plasmodium*-infected anopheline species are commonly found.

**Fig. 4:**
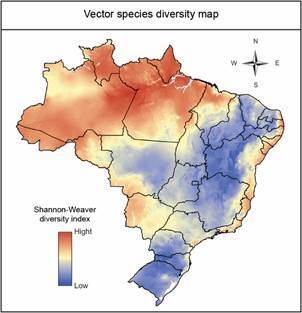
diversity of 28 Anophelinae species or putative species with potential role in malaria transmission, using Shannon-Wiener diversity index in Brazil.

Mapping geographical distribution of Anophelinae species in Brazil

The geographical distribution of species within the subfamily Anophelinae (Figs- 5 - 9) highlights their varying adaptability to ecological regions characterized by distinct biotic and abiotic factors, such as climate, hydrological features, and vegetation. While some species are generalists, thriving across multiple biomes, others display more specialized preferences [Supplementary data (Table I)]. For instance, *Ny. aquasalis* is closely associated with saline waters along Atlantic coast, and *Ny. rangeli* is primarily found in the Amazon region (Fig. 5A). Species within the Konderi Complex are distributed throughout the Amazon region, with *Ny. konderi* extending southwest into western Paraná State. *Ny. tadei* reaches as far as Mato Grosso do Sul State, extending to southern of the Pantanal region, *Ny*. *konderi* A is restricted to Amazon forest, *Ny*. *konderi* C is found in a specific area in the southern Pantanal (Fig. 5B, C). *Ny. oswaldoi* A and *Ny*. *oswaldoi* B are both found in the Amazon rainforest and considered potential vectors of *Plasmodium* spp. [Fig. 5D, F, Supplementary data (Table I)]. Additionally, specimens previously identified as *Ny. oswaldoi* or *Ny. oswaldoi* s.l. across the Amazon region may be *Ny. oswaldoi* A, likely due to misidentification (Fig. 5D-F). Among species distributed across Brazil’s major biomes, 26 have been collected exclusively within the Amazon rainforest, while 19 species have been reported solely in the Atlantic tropical rainforest. Within the Cerrado biome, four valid species - *An. tibiamaculatus*, *Lp. gilesi*, *Ny. albertoi*, and *Ny. untii* - have been documented exclusively, along with two potential new species, *Ny. parvus* Type 1 and *Ny. parvus* Type 2 [Supplementary data (Table I)]. Two species were recorded in forested areas within the Caatinga biome: *Ch. rozeboomi*, found in Londa, near Crato, Ceará, at an elevation of 500 meters. This species inhabits a wooded area in a transition zone among the Caatinga, Cerrado, and Atlantic tropical rainforest in the Chapada do Araripe.^92^ The second species, *Ny. ibiapabaensis*, was recently described from specimens collected at an altitude of 900 meters in the municipality of São Benedito.^93^ *Ny. janconnae*, a primary vector of human *Plasmodium*,^94^appears to be a habitat specialist, as it has been found exclusively within the Savanna (known locally as Lavrado) zone near Boa Vista in Roraima State. Among the habitat-specialist species, *Ch. fajardi*/*rozeboomi*, *Ke. laneana*, *Ny. antunesi* Type 1, *Ny. antunesi*, *Ny. lanei*, and *Ny. pristinus* have been recorded only in the Serra da Mantiqueira region in São Paulo State. Meanwhile, *Ny. antunesi* Types 2, 3, and 4 have been found in mountainous areas along the southern boundary of the Atlantic tropical rainforest. *Ny. antunesi* Type 2 has been collected in both the Serra da Mantiqueira and along the southern boundary of the Atlantic rainforest, while *Ny*. *pristinus* Type 1 has been identified in a mountainous area that is part of the Atlantic Forest biome, within the mountain range of the Tapiraí region in Vale do Ribeira, São Paulo State. Forty-one species from the genera *Anopheles*, *Chagasia*, *Nyssorhynchus*, and *Stethomyia* are generalist mosquitoes distributed across various Brazilian biomes. Understanding this distribution requires careful consideration of potential species misidentification, stemming from limited taxonomic and ecological research.

**Fig. 5:**
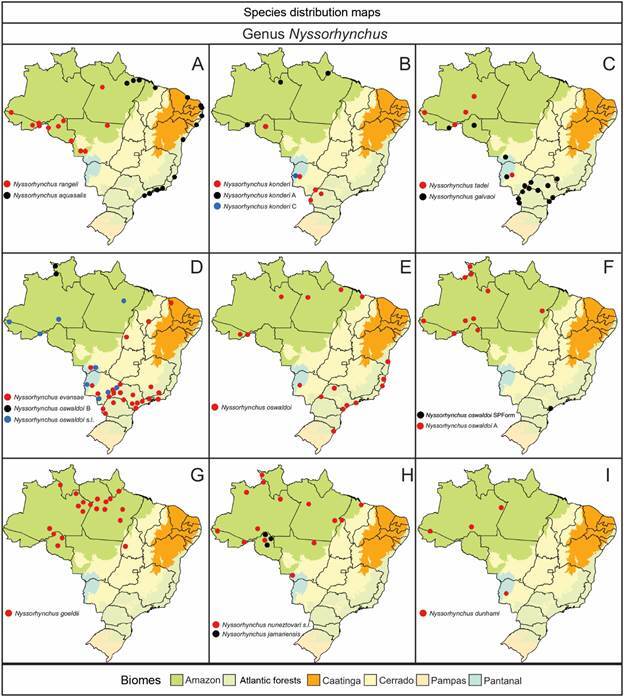
maps showing the distribution of the major Brazilian biomes, localities of collections of specimens of 16 species of the genus *Nyssorhynchus*. (A) *Nyssorhynchus rangeli* and *Nyssorhynchus aquasalis*; (B) *Nyssorhynchus konderi*, *Ny. konderi* A, *Ny. konderi* C; (C) *Nyssorhynchus tadei*, *Nyssorhynchus galvaoi*; (D) *Nyssorhynchus evansae*, *Nyssorhynchus oswaldoi* B, *Ny. oswaldoi* l.s.; (E) *Ny. oswaldoi*; (F) *Ny. oswaldoi* SPForm, *Ny. oswaldoi* A; (G) *Nyssorhynchus goeldii*; (H) *Nyssorhynchus nuneztovari*, *Nyssorhynchus jamariensis*; (I) *Nyssorhynchus dunhami*.

**Fig. 6:**
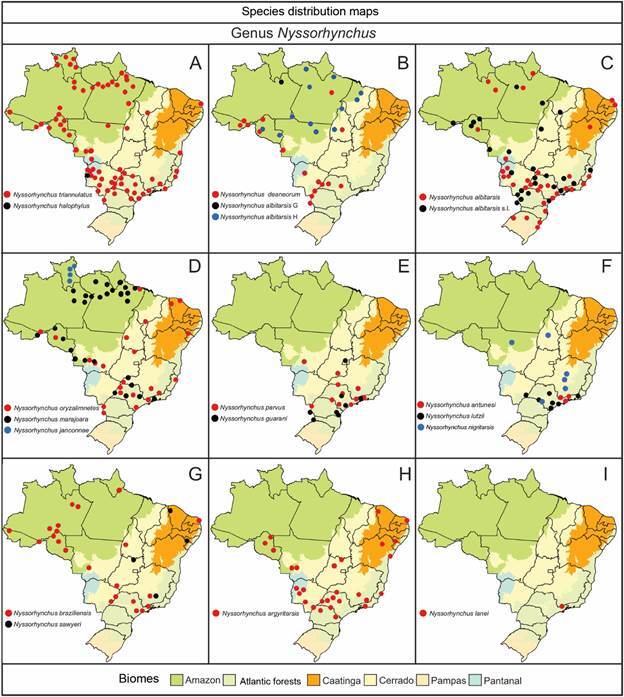
maps showing the distribution of the major Brazilian biomes, localities of collections of specimens of 18 species of the genus *Nyssorhynchus*. (A) *Nyssorhynchus triannulatus*, *Nyssorhynchus halophylus*; (B) *Nyssorhynchus deaneorum*, *Nyssorhynchus albitarsis* G. *Ny. albitarsis* H; (C) *Ny. albitarsis*, *Ny. albitarsis* l.s; (D) *Nyssorhynchus oryzalimnetes*, *Nyssorhynchus marajoara*, *Nyssorhynchus janconnae*; (E) *Nyssorhynchus parvus*, *Nyssorhynchus guarani*; (F) *Nyssorhynchus antunesi*, *Nyssorhynchus lutzii*, *Nyssorhynchus nigritarsis*; (G) *Nyssorhynchus braziliensis*, *Nyssorhynchus sawyeri*; (H) *Nyssorhynchus argyritarsis*; (I) *Nyssorhynchus lanei*.

**Fig. 7:**
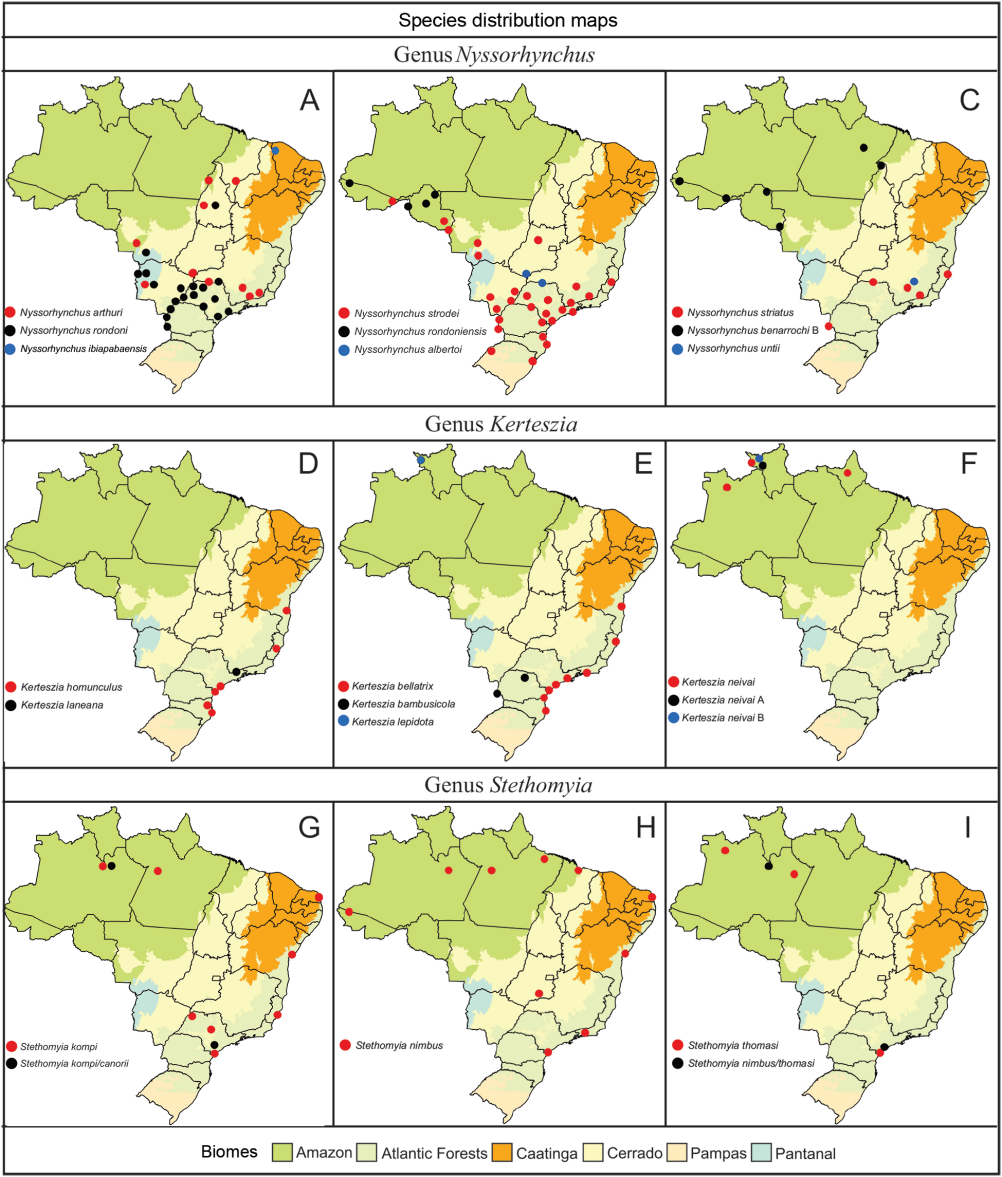
maps showing the distribution of the major Brazilian biomes, localities of collections of specimens of 22 species of the genera *Nyssorhynchus*, *Kerteszia*, *Stethomyia*. (A) *Nyssorhynchus arthuri*, *Nyssorhynchus rondoni*, *Nyssorhynchus ibiapabaensis*; (B) *Nyssorhynchus strodei*, *Nyssorhynchus rondoniensis*, *Nyssorhynchus albertoi*; (C) *Nyssorhynchus striatus*, *Nyssorhynchus benarrochi* B, *Nyssorhynchus untii*; (D) *Kerteszia homunculus*, *Kerteszia laneana*; (E) *Kerteszia belatrix*, *Kerteszia bambusicola*, *Kerteszia lepidota*; (F) *Kerteszia neivai*, *Ke. neivai* A, *Ke. neivai* B; (G) *Stethomyia kompi*, *Stethomyia kompi/canorii*; (H) *Stethomyia nimbus*; (I) *Stethomyia thomasi*; *Stethomyia nimbus/thomasi*.

**Fig. 8:**
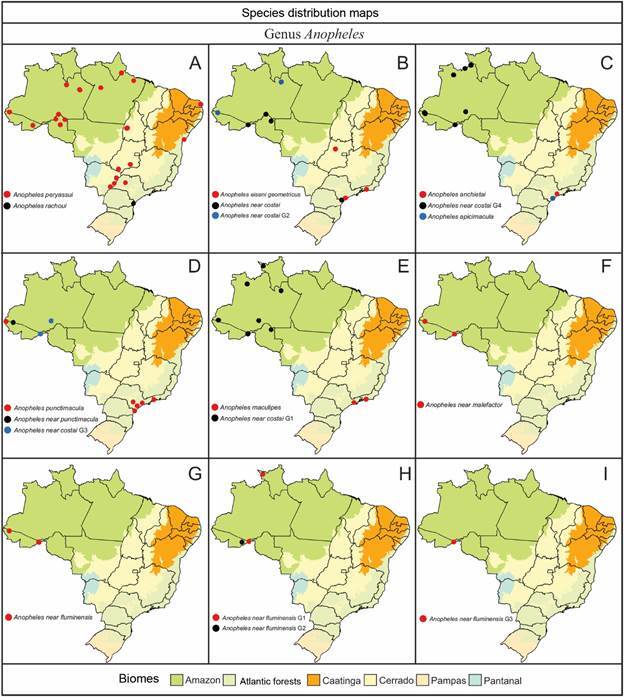
maps showing the distribution of the major Brazilian biomes, localities of collections of specimens of 18 species of the genus *Anopheles*. (A) *Anopheles peryassui*, *An. rachoui*; (B) *Anopheles eiseni geometricus*, *Anopheles* near *costai*, *Anopheles* near *costai* G2; (C) *Anopheles anchietai*, *Anopheles* near *costai* G4; *Anopheles apicimacula*; (D) *Anopheles puntimacula*, *Anopheles* near *punctimacula*, *Anopheles* near *costai* G3; (E) *Anopheles maculipes*, *Anopheles* near *costai* G1; (F) *Anopheles* near *malefactor*; (G) *Anopheles* near *fluminensis*; (H) *Anopheles* near *fluminensis* G1, *Anopheles* near *fluminensis* G2; (I) *Anopheles* near *fluminensis* G3.

**Fig. 9:**
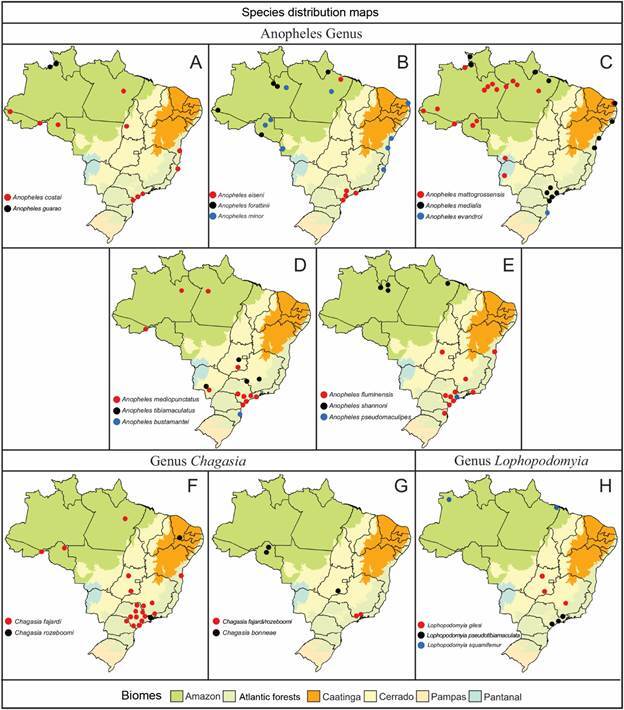
maps showing the distribution of the major Brazilian biomes, localities of collections of specimens of 21 species of the genera *Anopheles*, *Chagasia*, and *Lophopodomyia*. (A) *Anopheles costai*, *Anopheles guarao*; (B) *Anopheles eiseni*, *Anopheles forattini*, *Anopheles minor*; (C) *Anopheles mattogrossensis*, *Anopheles medialis*, *Anopheles evandroi*; (D) *Anopheles medipunctatus*, *Anopheles tibiamaculatus*, *Anopheles bustamantei*; (E) *Anopheles fluminensis*, *Anopheles shannoni*; *Anopheles pseudomaculipes*; (F) *Ch. fajardi*, *Ch. rozeboomi*; (G) *Ch. fajardi*/*rozeboomi*, *Ch*. *bonneae*; (H) *Lp. gilesi*, *Lp. pseudotibiamaculata*, *Lp. squamifemur*.

For example, *Ny. strodei*, initially classified as a single species with a broad distribution, is now recognized as a complex comprising at least seven distinct species.^93^ Similarly, species within the *Myzorhynchella* subgenus of *Nyssorhynchus* remain under-sampled, largely because locating and accessing their larval habitats in forested regions is challenging. Enhanced sampling efforts in the Atlantic tropical rainforest and Cerrado biomes have revealed ten species previously unknown to science, alongside five recognized species.^87^ The broad geographical distribution observed in certain species of the genera *Nyssorhynchus* and *Anopheles* may be partially attributed to their preference for permanent larval habitats such as river basins, fishponds, ponds, lakes, canals, river margins, floodplains, and swamps - habitats that are relatively accessible for field investigations.

Field sampling biases, which influence perceived species distribution, were initially noted by Neiva^1^ and remain relevant for understanding Anophelinae spatial distribution in Brazil. To address potential biases in anopheline species distribution, several factors should be considered. Sampling effort bias, for example, can skew species occurrence data, as intensive focus on particular habitats can lead to underrepresentation of species from less-sampled environments, thus distorting the overall knowledge of species ranges. Temporal bias affects data collection across seasons and years, impacting the detection of species with seasonal or cyclical population dynamics. Taxonomic bias also plays a role; certain species, such as *Ny. darlingi* and species within the Albitarsis Complex, receive more research attention, while others remain understudied. Detection bias is similarly significant, as species inhabiting specialized environments, such as phytotelmata (plant-associated water bodies), Amazonian igapó forest, small forest ponds, or debris-covered habitats, are often overlooked. Geographical bias may account for data scarcity in under-sampled biomes such as the Pantanal, Pampas, and Caatinga. Additionally, environmental changes driven by habitat destruction and fragmentation alter mosquito communities, potentially misrepresenting species distributions, as seen in the Cerrado biome.

Vector surveillance for malaria elimination in Brazil

The World Malaria Report highlights a 27% reduction of global malaria incidence and mortality from 2000 to 2015.^95^ In subsequent years, progress has slowed, with a 2% reduction since 2015. Because of the stagnation of malaria control progress, the world faces a critical moment to prevent further setbacks, thus malaria control requires intensified and multifaceted interventions.^75^ The control for elimination strategies needs to address socioeconomic and environmental determinants of transmission dynamics,^96^ in addition to factors associated with malaria protozoans, and mosquito vectors. At a certain level, innovative technologies can provide alternatives for malaria and vector control. For instance, genetically modified mosquitoes that are refractory to *Plasmodium* infection can help to decrease the density of infective mosquitoes in a transmission setting,^97^ while geography and geospatial analysis will be important for monitoring malaria and the impacts of control strategies.^69^

Malaria in Brazil has decreased over the years, but it persists in remote areas, particularly in the Amazon region, where aggressive land occupation, extensive deforestation, and human migration in and out of these areas contribute to ongoing transmission. The most affected and vulnerable areas are mining sites, indigenous lands, and rural regions, where limited access and the sustainability of control measures are significant challenges. Additionally, deforestation and proximity to forested environments increase human exposure to mosquito vectors, as observed in rural settlements in the Amazon.^61^ In 2023, *P. falciparum* and mixed malaria cases significantly increased in indigenous and mining areas. Delays in diagnosis and treatment exacerbate transmission and case severity. Ensuring accurate treatment data, continuous education for healthcare workers, and targeted health education for at-risk groups is essential to raise awareness and promote timely treatment.^98^

Monitoring the distribution of mosquito species that are vectors of *Plasmodium* spp. poses significant challenges for vector surveillance programs of countries where malaria is endemic.^71^ Environmental changes, such as deforestation, urbanization, and agricultural expansion, can alter the distribution of habitats of Anophelinae mosquitoes, creating new habitats or diminishing existing ones.^99^ These dynamics make it difficult to predict and track mosquito populations over time, particularly in areas experiencing ecological shifts.^69^ Additionally, many Anophelinae species are difficult to differentiate morphologically, which complicates identification efforts in the field and increases the risk of misidentification in surveillance activities.^86^

Another challenge is the limited availability of comprehensive, up-to-date distribution data for many Anophelinae species.^24^ Although field data from multiple collections across Brazilian territories provide valuable information, there are still gaps in the knowledge of species distributions. These gaps can hinder the development of effective control strategies, as vector surveillance must be tailored to local ecological and epidemiological contexts. Moreover, many areas with high malaria transmission risk, particularly remote or understudied regions, lack adequate surveillance infrastructure, further complicating efforts to monitor species and implement timely interventions.^100^ To address these challenges, it is crucial to integrate multiple approaches of vector surveillance, including molecular techniques for species identification, geographic information systems (GIS) for mapping species distributions, and ecological niche modeling to predict potential vector habitats under different environmental scenarios.^101^ ^,^ ^102^ Enhancing surveillance systems with these advanced tools will improve the accuracy of mosquito monitoring and support more effective interventions aimed at eliminating malaria transmission in endemic countries and in Brazil, as well.

In conclusion

Despite progress in understanding the distribution of Anophelinae species in Brazil since Neiva’s 1909 study, significant gaps remain, particularly in remote and ecologically complex biomes such as the Amazon, Cerrado, Pantanal, Pampas, and Caatinga. Limited data on the distribution, ecological preferences, and population dynamics of malaria vectors, especially species beyond *Ny. darlingi* and *Ke. cruzii*, from the *Nyssorhynchus*, *Kerteszia*, and *Anopheles* genera, hinder the development of targeted control strategies. These knowledge gaps impede efforts to accurately map transmission risk, predict vector behavior in diverse environments, design effective vector control interventions, and implement effective public health responses. For instance, sylvatic species capable of transmitting *Plasmodium* to human hosts in forested areas beyond human dwellings may continue to act as reservoirs, increasing the risk of outbreaks in both endemic and non-endemic areas. Addressing these knowledge gaps about vector species across all Brazilian biomes is essential for improving vector surveillance and control efforts, ultimately contributing to malaria elimination in Brazil.
