# Supplementary material for: Distribution of Anophelinae (Diptera: Culicidae) and challenges for malaria elimination in Brazil
Source: Mem Inst Oswaldo Cruz. 2025 Feb 24;120:e240247. doi: 10.1590/0074-02760240247 (PMC11852321; doi:10.1590/0074-02760240247)
Supplement: Supplementary file 1 [file 1678-8060-mioc-120-e240247-s.pdf]

Data collection and modeling distribution

The data on Anophelinae species from the Coleção Entomológica de Referência, Faculdade de Saúde Pública (CER-FSP), along with data from field collections in Brazilian territory and published literature records focused on taxonomic studies, were retrieved and used for distribution analyses and the creation of geographical distribution maps (Table I). The criterion for a municipality or location inclusion in the spatial database was the confirmation of the presence of a species. Geographical coordinates were either the centroid of the municipality where the species was recorded or the exact latitude and longitude of the collection sites. Geographical distribution maps were generated using the software QGIS version 2.6.1 [QGIS Development Team. Open-Source Geospatial Foundation (OSGeo)]. Additionally, for the twenty-eight vector species, the MaxEnt software(1) was employed for modeling species distributions by maximum entropy of climatic variables. The climatic variables, including annual mean temperature (ºC = Celsius degree), mean diurnal range, isothermality, temperature seasonality. maximum temperature of the warmest month, minimum temperature of the coldest month, temperature annual range, annual precipitation, precipitation of the wettest month, precipitation of the driest month, precipitation seasonality, and relative humidity, were analyzed in relation to the presence of all vector species. These variables were selected because they affect the life cycles and bionomics of mosquitoes. The climate data were available at the WorldClim database — Global Climate Data,(2) at a resolution of 30 arc seconds (approximately 1 km). A principal components analysis (PCA) was conducted to pre-select the most representative climatic variables for these species studied. Following the PCA, the four most representative eigenvectors were selected for use in the modeling analysis. The maximum entropy distribution model (MaxEnt)(1) was chosen, as it demonstrated the best performance compared to other species distribution models.(3)

After modeling the potential geographic distribution of vector species, a map illustrating the diversity distribution of these anophelines was generated. The Shannon-Wiener diversity index(4) was applied to perform this analysis. This index accounts for the proportion of each species within the community, implying that species occurrences are not equally independent of sample size. This relationship is represented by the expression:

$$H' = -\sum_{(i=1)}^{n} pi.Ln(pi)$$

Where: *H'* represents the diversity index, and *pi* is the proportional abundance of each species in the sample.

This formula reflects the combined influence of species richness and evenness, providing a nuanced measure of diversity across the mapped geographic range. To calculate the Shannon-Wiener index, the results of maximum entropy modeling were adapted to estimate the relative abundance of each mosquito species. Using this approach was possible to produce a diversity map of anopheline vector species.

RESULTS

In the PCA analysis, the first two components (PCA1 and PCA2) accounted for the most significant eigenvalues. These components explained the variation for all vector species analyzed (Table II). The PCA showed the most significant variables for each anopheline vector species studied (Table III).

CC BY

TABLE I

Summary of valid species included in this study, listing genus, subgenus, series, collection state, and *Plasmodium* vector status. Unnamed species identified by COI barcode are also included and marked accordingly

Genus	Subgenus	Series	Species	State	Status *Plasmodium* vector
Anopheles	*Anopheles*	Arribalzagia	*Anopheles anchietai*	SP	unknown
Anopheles	*Anopheles*	Arribalzagia	*Anopheles apicimacula*	SP	unknown
Anopheles	*Anopheles*	Arribalzagia	*Anopheles bustamantei*	SC	unknown
Anopheles	*Anopheles*	Arribalzagia	*Anopheles costai*	AC, AM, BA, ES, PA, RO, SP, TO	unknown
Anopheles	*Anopheles*	Anopheles	*Anopheles eiseni*	PA, SP	unknown
Anopheles	*Anopheles*	Anopheles	*Anopheles eiseni geometricus*	GO, RJ, SP	unknown
Anopheles	*Anopheles*	Arribalzagia	*Anopheles evandroi*	SC	unknown
Anopheles	*Anopheles*	Arribalzagia	*Anopheles fluminensis*	BA, GO, MG, RJ, SC, SP	unknown
Anopheles	*Anopheles*	Arribalzagia	*Anopheles forattinii*	AM, AP, RO	poorly known in Brazil
Anopheles	*Anopheles*	Arribalzagia	*Anopheles guarao*	AM	unknown
Anopheles	*Anopheles*	Arribalzagia	*Anopheles maculipes*	RJ, SP	unknown
Anopheles	*Anopheles*	Arribalzagia	*Anopheles mattogrossensis*	AC, AM, MS, MT, PA, RN, RO	poorly known in Brazil
Anopheles	*Anopheles*	Arribalzagia	*Anopheles medialis*	AL, AM, AP, BA, PA, RN, RR, SP	poorly known in Brazil
Anopheles	*Anopheles*	Arribalzagia	*Anopheles mediopunctatus*	AC, AM, GO, MS, PA, SP	unknown
Anopheles	*Anopheles*	Arribalzagia	*Anopheles minor*	AM, BA, ES, MT, PA, RN, RO	unknown
Anopheles	*Anopheles*	Arribalzagia	*Anopheles* near *costai*	AC, AM, RO, SP	unknown
Anopheles	*Anopheles*	Arribalzagia	*Anopheles* near *costai* G1	AC, AM, RO, RR	found infected in Loreto, Peru, potential vector in Brazil
Anopheles	*Anopheles*	Arribalzagia	*Anopheles* near *costai* G2	AC, AM	unknown
Anopheles	*Anopheles*	Arribalzagia	*Anopheles* near *costai* G3	AC, AM	unknown
Anopheles	*Anopheles*	Arribalzagia	*Anopheles* near *costai* G4	AC, AM	unknown
Anopheles	*Anopheles*	Arribalzagia	*Anopheles* near *fluminensis*	AC	unknown
Anopheles	*Anopheles*	Arribalzagia	*Anopheles* near *fluminensis* G1	AC, RR	unknown
Anopheles	*Anopheles*	Arribalzagia	*Anopheles* near *fluminensis* G2	AC	unknown
Anopheles	*Anopheles*	Arribalzagia	*Anopheles* near *fluminensis* G3	AC	unknown
Anopheles	*Anopheles*	Arribalzagia	*Anopheles* near *malefactor*	AC, AM	unknown
Anopheles	*Anopheles*	Arribalzagia	*Anopheles* near *punctimacula*	AC	unknown
Anopheles	*Anopheles*	Arribalzagia	*Anopheles peryassui*	AC, AM, AP, BA, GO, MS, PA, RN, RO, SP, TO	found infected in locations in Amazonas State, Brazil
Anopheles	*Anopheles*	Arribalzagia	*Anopheles pseudomaculipes*	SP	unknown
Anopheles	*Anopheles*	Arribalzagia	*Anopheles punctimacula*	AC, RJ, SP	unknown
Anopheles	*Anopheles*	Arribalzagia	*Anopheles rachoui*	PR	unknown
Anopheles	*Anopheles*	Arribalzagia	*Anopheles shannoni*	AM, PA	unknown
Anopheles	*Anopheles*	Anopheles	*Anopheles tibiamaculatus*	GO, MG, MS, SP	unknown
Chagasia			*Chagasia bonneae*	GO, RO	unknown
Chagasia			*Chagasia fajardi*	AC, BA, GO, MG, PA, PR, RJ, RO, SP	unknown
Chagasia			*Chagasia fajardi/rozebomi*	RJ, SP	unknown
Chagasia			*Chagasia rozeboomi*	CE, SP	unknown
Kerteszia			*Kerteszia bambusicola*	PR	unknown
Kerteszia			*Kerteszia bellatrix*	BA, ES, PR, RJ, SC, SP	local vector in southeastern Atlantic Forest
Kerteszia			*Kerteszia cruzii*	BA, ES, PE, PR, RJ, RS, SC, SE, SP	local vector in southeastern Atlantic Forest

Genus	Subgenus	Series	Species	State	Status *Plasmodium* vector
Kerteszia			*Kerteszia homunculus*	BA, ES, SC, SP	local vector in southeastern Atlantic Forest
Kerteszia			*Kerteszia laneana*	SP	unknown
Kerteszia			*Kerteszia lepidota*	RR	vector in Colombia, potential vector in Brazil
Kerteszia			*Kerteszia neivai*	AM, AP, RR	vector in locations on Pacific Coast, Colombia, potential vector in Brazil
Kerteszia			*Kerteszia neivai* A	RR	unknown
Kerteszia			*Kerteszia neivai* B	RR	unknown
Lophopodomyia			*Lophopodomyia gilesi*	GO, MG	unknown
Lophopodomyia			*Lophopodomyia pseudotibimaculata*	SP	unknown
Lophopodomyia			*Lophopodomyia squamifemur*	AM, PA	unknown
Nyssorhynchus	*Nyssorhynchus*	Oswaldoi	*Nyssorhynchus albertoi*	GO, MG	unknown
Nyssorhynchus	*Nyssorhynchus*	Albitarsis	*Nyssorhynchus albitarsis*	AM, AP, BA, ES, MG, MS, MT, PR, RJ, RN, RO, RS, SC, SP	unknown
Nyssorhynchus	*Nyssorhynchus*	Albitarsis	*Nyssorhynchus albitarsis* G	AM	Foley et al. (2014) potential vector in Brazil
Nyssorhynchus	*Nyssorhynchus*	Albitarsis	*Nyssorhynchus albitarsis* H	PA, MA, TO, MT, RO	Foley et al. (2014) potential vector in Brazil
Nyssorhynchus	*Nyssorhynchus*	Albitarsis	*Nyssorhynchus albitarsis* s.l.	AC, ES, GO, MA, MG, MS, MT, PA, PR, RO, SP, TO	Sinka et al. (2012) considered a dominant vector in Brazil
Nyssorhynchus	*Myzorhynchella*		*Nyssorhynchus antunesi*	RJ, SP	unknown
Nyssorhynchus	*Myzorhynchella*		*Nyssorhynchus antunesi* Type 1	SP	unknown
Nyssorhynchus	*Myzorhynchella*		*Nyssorhynchus antunesi* Type 2	RJ, RS	unknown
Nyssorhynchus	*Myzorhynchella*		*Nyssorhynchus antunesi* Type 3	RS	unknown
Nyssorhynchus	*Myzorhynchella*		*Nyssorhynchus antunesi* Type 4	RS	unknown
Nyssorhynchus	*Nyssorhynchus*	Oswaldoi	*Nyssorhynchus aquasalis*	AL, BA, CE, ES, MA, PA, PE, PB, RJ, RN, SP	dominant vector across Atlantic Coast
Nyssorhynchus	*Nyssorhynchus*	Argyritarsis	*Nyssorhynchus argyritarsis*	BA, CE, ES, GO, MG, MS, MT, PR, RJ, RN, RO, SE, SP	unknown
Nyssorhynchus	*Nyssorhynchus*	Oswaldoi	*Nyssorhynchus arthuri*	GO, MA, MG, MS, MT, TO	unknown
Nyssorhynchus	*Nyssorhynchus*	Oswaldoi	*Nyssorhynchus benarrochi* B	AC, PA, RO, TO	found infected in Pará State, Brazil and several provinces in Peru
Nyssorhynchus	*Nyssorhynchus*	Argyritarsis	*Nyssorhynchus braziliensis*	AC, AM, AP, GO, MG, MS, MT, RN, RO, SP, TO	found infected in locations across Brazilian Amazon
Nyssorhynchus	*Nyssorhynchus*	Argyritarsis	*Nyssorhynchus darlingi*	AC, AL, AM, AP, BA, CE, ES, GO, MG, MA, MS, MT, PA, PI, PR, RJ, RO, RR, SE, SP, TO	dominant vector in Brazilian Amazon
Nyssorhynchus	*Nyssorhynchus*	Albitarsis	*Nyssorhynchus deaneorum*	AC, MS, MT, PA, PR, RO, SP, TO	local malaria vector in Acre State, Brazil
Nyssorhynchus	*Nyssorhynchus*	Oswaldoi	*Nyssorhynchus dunhami*	AC, AM, MS	found infected in Iquitos, Peru, potential vector in Brazil
Nyssorhynchus	*Nyssorhynchus*	Oswaldoi	*Nyssorhynchus evansae*	CE, MA, MG, MS, MT, PR, RJ, RS, TO, SP	unknown
Nyssorhynchus	*Nyssorhynchus*	Oswaldoi	*Nyssorhynchus galvaoi*	AC, MG, MS, MT, PR, RO, SP	unknown
Nyssorhynchus	*Nyssorhynchus*	Oswaldoi	*Nyssorhynchus goeldii*	AM, AP, PA, RO, TO	found infected in locations across Brazilian Amazon
Nyssorhynchus	*Myzorhynchella*		*Nyssorhynchus guarani*	DF, PR, RJ, SC, SP	unknown
Nyssorhynchus	*Nyssorhynchus*	Triannulatus	*Nyssorhynchus halophylus*	MS	unknown
Nyssorhynchus	*Nyssorhynchus*	Oswaldoi	*Nyssorhynchus ibiapabaensis*	CE	unknown
Nyssorhynchus	*Nyssorhynchus*	Oswaldoi	*Nyssorhynchus jamariensis*	RO	unknown
Nyssorhynchus	*Nyssorhynchus*	Albitarsis	*Nyssorhynchus janconnae*	RR	primary vector in locations in Roraima State, Brazil
Nyssorhynchus	*Nyssorhynchus*	Oswaldoi	*Nyssorhynchus konderi*	MS, PR, RO	unknown
Nyssorhynchus	*Nyssorhynchus*	Oswaldoi	*Nyssorhynchus konderi* A	AC, AM, AP	unknown
Nyssorhynchus	*Nyssorhynchus*	Oswaldoi	*Nyssorhynchus konderi* C	MS	unknown
Nyssorhynchus	*Nyssorhynchus*	Argyritarsis	*Nyssorhynchus lanei*	SP	unknown
Nyssorhynchus	*Myzorhynchella*		*Nyssorhynchus lutzii*	MS, PR, RJ, SP	unknown
Nyssorhynchus	*Myzorhynchella*		*Nyssorhynchus lutzii* Type 1	RS	unknown

Genus	Subgenus	Series	Species	State	Status *Plasmodium* vector
Nyssorhynchus	*Myzorhynchella*		*Nyssorhynchus lutzii* Type 2	RS	unknown
Nyssorhynchus	*Nyssorhynchus*	Albitarsis	*Nyssorhynchus marajoara*	AC, AM, AP, MG, MT, PA, RJ, RO, SP	dominant vector across Brazilian Amazon
Nyssorhynchus	*Myzorhynchella*		*Nyssorhynchus nigritarsis*	GO, MG, MT, PR, TO	unknown
Nyssorhynchus	*Nyssorhynchus*	Oswaldoi	*Nyssorhynchus nuneztovari* s.l.	AC, AM, MA, MT, PA, RO, RR, TO	primary vector in Venezuela and Colombia
Nyssorhynchus	*Nyssorhynchus*	Albitarsis	*Nyssorhynchus oryzalimnetes*	AC, CE, ES, GO, MA, MG, MT, PA, PR, RO, SE, SP, TO	unknown
Nyssorhynchus	*Nyssorhynchus*	Oswaldoi	*Nyssorhynchus oswaldoi*	AC, AM, BA, ES, MA, MS, PA, RS, SP	unknown
Nyssorhynchus	*Nyssorhynchus*	Oswaldoi	*Nyssorhynchus oswaldoi* A	AC, AM, PA, RO, RR	found infected in Rondônia State, Brazil
Nyssorhynchus	*Nyssorhynchus*	Oswaldoi	*Nyssorhynchus oswaldoi* B	AM, RR	potential vector in Putumayo, Colombia
Nyssorhynchus	*Nyssorhynchus*	Oswaldoi	*Nyssorhynchus oswaldoi* s.l.	AC, AM, MS, MT, PA	unknown
Nyssorhynchus	*Nyssorhynchus*	Oswaldoi	*Nyssorhynchus oswaldoi* SPForm	SP	unknown
Nyssorhynchus	*Myzorhynchella*		*Nyssorhynchus parvus*	GO, MG, MT, PR, RJ, SP	unknown
Nyssorhynchus	*Myzorhynchella*		*Nyssorhynchus parvus* Type1	DF, MG, MT	unknown
Nyssorhynchus	*Myzorhynchella*		*Nyssorhynchus parvus* Type2	DF	unknown
Nyssorhynchus	*Myzorhynchella*		*Nyssorhynchus pristinus*	SP	unknown
Nyssorhynchus	*Myzorhynchella*		*Nyssorhynchus pristinus* Type1	SP	unknown
Nyssorhynchus	*Nyssorhynchus*	Oswaldoi	*Nyssorhynchus rangeli*	AC, AM, MT, PA, RO	found infected in Acre State, Brazil
Nyssorhynchus	*Nyssorhynchus*	Oswaldoi	*Nyssorhynchus rondoni*	MG, MS, MT, PR, SP, TO	unknown
Nyssorhynchus	*Nyssorhynchus*	Oswaldoi	*Nyssorhynchus rondoniensis*	AC, RO	found infected in Rondônia State, Brazil
Nyssorhynchus	*Nyssorhynchus*	Argyritarsis	*Nyssorhynchus sawyeri*	CE, GO, MG, SE	unknown
Nyssorhynchus	*Nyssorhynchus*	Oswaldoi	*Nyssorhynchus striatus*	ES, MG, PR	unknown
Nyssorhynchus	*Nyssorhynchus*	Oswaldoi	*Nyssorhynchus strodei*	AC, ES, GO, MG, MS, MT, PR, RO, RS, SC, SP	found infected in locations in São Paulo State, Brazil
Nyssorhynchus	*Nyssorhynchus*	Oswaldoi	*Nyssorhynchus tadei*	AC, AM, MS	found infected in Acre State, Brazil
Nyssorhynchus	*Nyssorhynchus*	Triannulatus	*Nyssorhynchus triannulatus*	AC, AM, AP, BA, ES, GO, MA, MG, MS, MT, PA, PR, RJ, RN, RO, RR, SP, TO	found infected in Acre State, Brazil
Nyssorhynchus	*Nyssorhynchus*	Oswaldoi	*Nyssorhynchus untii*	MG	unknown
Stethomyia			*Stethomyia kompi*	AM, BA, ES, PA, RN, SP	unknown
Stethomyia			*Stethomyia kompi/canorii*	AM, SP	unknown
Stethomyia			*Stethomyia nimbus*	AC, AM, BA, GO, MA, PA, RJ, RN, SP	unknown
Stethomyia			*Stethomyia nimbus/thomasi*	AM, SP	unknown
Stethomyia			*Stethomyia thomasi*	AM, SP	unknown

AC, Acre; AL, Alagoas; AM, Amazonas; AP, Amapá; BA, Bahia; CE, Ceará; DF, Distrito Federal; ES, Espírito Santo; GO, Goiás; MA, Maranhão; MG, Minas Gerais; MS, Mato Grosso do Sul; MT, Mato Grosso; PA, Pará; PB, Paraíba; PE, Pernambuco; PI, Piauí; PR, Paraná; RJ, Rio de Janeiro; RN, Rio Grande do Norte; RO, Rondônia; RR, Roraima; RS, Rio Grande do Sul; SC, Santa Catarina; SE, Sergipe; SP, São Paulo; TO, Tocantins.

TABLE II

Results of the principal component analysis (PCA) of climate variables

Species	PCA results
Anopheles forattinii	72.3
Anopheles mattogrossensis	69.0
Anopheles medialis	73.4
Anopheles peryassui	80.5
Kerteszia bellatrix	66.3
Kerteszia cruzii	62.9
Kerteszia homunculus	77.9
Kerteszia lepidota	66.4
Kerteszia neivai	63.3
Nyssorhynchus albitarsis G	68.8
Nyssorhynchus albitarsis H	72.9
Nyssorhynchus aquasalis	75.3
Nyssorhynchus benarrochi B	70.6
Nyssorhynchus braziliensis	79.1
Nyssorhynchus darlingi	77.4
Nyssorhynchus deaneorum	84.9
Nyssorhynchus dunhami	75.1
Nyssorhynchus goeldii	65.5
Nyssorhynchus janconnae	82.9
Nyssorhynchus marajoara	79.1
Nyssorhynchus nuneztovari	68.9
Nyssorhynchus oswaldoi A	65.5
Nyssorhynchus oswaldoi B	72.3
Nyssorhynchus rangeli	79.3
Nyssorhynchus rondoniensis	80.3
Nyssorhynchus strodei	74.5
Nyssorhynchus tadei	76.3
Nyssorhynchus triannulatus	78.0

TABLE III

Results of the principal component analysis (PCA). The red number show the variables with the highest scores using eigenvectors

Species	Eigenvectors	Annual mean temperature	Annual precipitation	Precipitation of wettest month	Precipitation of driest month	Precipitation seasonality	Mean diurnal range	Isothermality	Temperature seasonality	Max temperature of warmest month	Min temperature of coldest month	Temperature annual range	Humidity relative
Anopheles forattinii	PC1	0.109	-0.334	-0.003	-0.362	0.372	0.326	-0.195	0.297	0.317	-0.161	0.369	-0.335
	PC2	0.535	0.077	0.243	0.127	0.021	-0.282	-0.230	-0.018	0.357	0.510	-0.180	-0.283
Anopheles mattogrossensis	PC1	0.308	0.412	0.223	0.395	-0.314	-0.131	0.258	-0.330	0.193	0.340	-0.276	0.099
	PC2	-0.323	0.218	0.159	-0.017	-0.057	0.435	0.390	-0.321	-0.122	-0.336	0.315	0.385
Anopheles medialis	PC1	0.366	0.327	0.284	0.171	-0.072	-0.130	0.324	-0.357	0.289	0.374	-0.351	0.220
	PC2	0.082	-0.072	0.211	-0.527	0.623	0.348	0.216	-0.087	0.251	0.030	0.095	-0.171
Anopheles peryassui	PC1	0.333	0.346	0.289	0.214	-0.125	-0.245	0.311	-0.336	0.011	0.354	-0.352	0.326
	PC2	0.162	0.026	0.180	-0.447	0.502	0.372	0.261	-0.203	0.479	0.019	0.104	0.031
Kerteszia bellatrix	PC1	0.404	-0.222	-0.292	0.096	-0.205	-0.205	0.275	-0.320	0.328	0.415	-0.352	-0.164
	PC2	-0.031	-0.495	-0.314	-0.425	0.260	0.502	0.242	-0.131	0.091	-0.078	0.214	0.138
Kerteszia cruzii	PC1	0.436	0.009	0.138	-0.203	0.213	-0.248	0.118	-0.296	0.339	0.450	-0.362	-0.177
	PC2	-0.098	-0.246	-0.002	-0.409	0.358	0.315	0.454	-0.362	-0.140	-0.073	-0.016	0.156
Kerteszia homunculus	PC1	0.261	0.420	0.315	0.213	-0.012	-0.366	-0.319	0.171	0.342	0.220	-0.131	-0.403
	PC2	-0.363	0.127	0.263	-0.200	0.324	0.240	-0.273	0.363	0.071	-0.397	0.432	-0.157
Kerteszia lepidota	PC1	-0.111	-0.376	-0.175	-0.380	0.346	0.395	0.095	0.186	0.241	-0.310	0.392	-0.209
	PC2	0.565	-0.120	0.143	-0.174	0.198	-0.073	0.211	-0.246	0.446	0.422	-0.167	-0.249
Kerteszia neivai	PC1	-0.160	-0.385	-0.119	-0.389	0.347	0.411	0.114	0.208	0.123	-0.317	0.408	-0.181
	PC2	0.446	-0.048	0.318	-0.238	0.313	0.033	0.364	-0.217	0.432	0.390	-0.144	-0.047
Nyssorhynchus albitarsis G	PC1	0.056	-0.323	0.325	-0.391	0.416	-0.048	-0.358	0.370	0.120	-0.050	0.224	-0.356
	PC2	0.509	0.306	0.264	0.165	-0.130	0.032	-0.061	0.089	0.517	0.494	0.094	0.024
Nyssorhynchus albitarsis H	PC1	-0.123	-0.338	-0.181	-0.333	0.349	0.337	-0.173	0.284	0.279	-0.296	0.354	-0.301
	PC2	0.541	-0.064	0.386	-0.076	0.226	-0.237	-0.239	0.217	0.305	0.388	-0.169	-0.255
Nyssorhynchus aquasalis	PC1	0.391	-0.043	0.145	-0.304	0.352	-0.072	0.359	-0.379	0.215	0.384	-0.321	-0.181
	PC2	-0.023	0.542	0.475	0.325	-0.134	-0.135	0.175	-0.179	-0.272	0.086	-0.204	0.391
Nyssorhynchus benarrochi B	PC1	-0.172	-0.315	-0.043	-0.361	0.338	0.388	-0.148	0.239	0.248	-0.341	0.389	-0.258
	PC2	0.528	0.011	0.416	0.000	0.234	-0.020	0.033	-0.216	0.466	0.300	-0.029	-0.370
Nyssorhynchus braziliensis	PC1	0.327	0.354	0.281	0.118	-0.133	-0.185	0.353	-0.359	0.186	0.362	-0.352	0.282
	PC2	-0.214	0.008	-0.156	0.510	-0.467	-0.419	-0.135	0.148	-0.416	-0.055	-0.159	0.187
Nyssorhynchus darlingi	PC1	0.315	0.297	0.272	0.148	-0.071	-0.196	0.288	-0.321	0.063	0.361	-0.337	0.209
	PC2	0.235	-0.140	0.087	-0.377	0.450	0.349	0.217	-0.160	0.434	0.057	0.150	-0.333
Nyssorhynchus deaneorum	PC1	0.319	0.233	0.331	-0.254	0.259	-0.127	0.363	-0.362	0.132	0.359	-0.353	0.226
	PC2	0.220	-0.379	-0.067	-0.356	0.333	0.383	-0.077	-0.001	0.482	0.039	0.133	-0.402
Nyssorhynchus dunhami	PC1	-0.272	-0.318	-0.268	-0.222	0.155	0.294	-0.307	0.354	0.212	-0.345	0.361	-0.283
	PC2	0.215	0.162	-0.025	0.515	-0.487	-0.170	-0.264	0.196	0.386	0.050	0.072	-0.361
Nyssorhynchus goeldii	PC1	0.393	0.098	0.075	0.315	-0.224	-0.418	-0.249	0.163	0.230	0.418	-0.349	-0.262
	PC2	0.109	-0.397	-0.260	-0.192	0.261	0.099	-0.369	0.419	0.318	-0.106	0.307	-0.360

Species	Eigenvectors	Annual mean temperature	Annual precipitation	Precipitation of wettest month	Precipitation of driest month	Precipitation seasonality	Mean diurnal range	Isothermality	Temperature seasonality	Max temperature of warmest month	Min temperature of coldest month	Temperature annual range	Humidity relative
Nyssorhynchus janconnae	PC1	-0.155	0.331	-0.053	0.360	-0.375	0.337	0.370	-0.378	-0.161	-0.125	-0.120	0.380
	PC2	0.501	0.247	0.386	0.160	-0.067	0.057	0.059	-0.040	0.497	0.500	-0.010	0.049
Nyssorhynchus marajoara	PC1	-0.295	-0.335	-0.268	-0.231	0.169	0.279	-0.339	0.345	-0.114	-0.350	0.354	-0.275
	PC2	0.351	-0.015	0.133	-0.391	0.395	0.309	0.151	-0.138	0.580	0.118	0.092	-0.231
Nyssorhynchus nuneztovari	PC1	-0.119	-0.335	-0.129	-0.302	0.285	0.341	-0.232	0.287	0.290	-0.344	0.382	-0.290
	PC2	0.245	0.126	0.588	-0.285	0.400	0.087	0.274	-0.340	0.247	0.152	-0.019	-0.230
Nyssorhynchus oswaldoi A	PC1	-0.211	-0.355	-0.127	-0.373	0.384	0.396	0.038	0.257	0.078	-0.362	0.385	-0.131
	PC2	0.421	-0.115	0.105	0.113	-0.029	-0.041	-0.393	0.228	0.564	0.187	0.134	-0.454
Nyssorhynchus oswaldoi B	PC1	-0.179	-0.300	0.214	-0.383	0.388	0.380	0.009	0.286	0.214	-0.294	0.395	-0.142
	PC2	0.475	-0.277	-0.031	-0.035	0.080	-0.167	-0.335	-0.055	0.468	0.352	-0.035	-0.450
Nyssorhynchus rangeli	PC1	-0.205	-0.239	-0.035	-0.243	0.264	0.353	-0.277	0.326	0.280	-0.351	0.380	-0.345
	PC2	0.223	0.368	0.534	-0.399	0.371	0.097	0.202	-0.306	0.258	0.102	0.013	-0.110
Nyssorhynchus rondoniensis	PC1	-0.066	-0.357	-0.348	0.237	-0.242	-0.044	-0.381	0.383	0.226	-0.260	0.299	-0.362
	PC2	-0.224	0.029	0.204	-0.363	0.397	0.559	-0.024	-0.018	0.203	-0.356	0.363	-0.068
Nyssorhynchus strodei	PC1	-0.335	-0.036	-0.274	0.341	-0.335	-0.235	-0.363	0.366	-0.243	-0.313	0.198	0.256
	PC2	0.109	0.532	0.165	0.214	-0.143	-0.455	-0.028	-0.106	-0.099	0.305	-0.471	0.258
Nyssorhynchus tadei	PC1	-0.276	-0.317	-0.259	-0.215	0.133	0.289	-0.316	0.352	0.221	-0.343	0.357	-0.303
	PC2	0.344	-0.053	0.378	-0.373	0.640	-0.058	-0.029	-0.052	0.337	0.190	-0.063	-0.162
Nyssorhynchus triannulatus	PC1	0.345	0.337	0.320	0.107	0.040	-0.210	0.345	-0.365	0.178	0.373	-0.359	0.237
	PC2	0.158	-0.084	0.116	-0.501	0.515	0.386	0.152	-0.132	0.387	0.003	0.150	-0.277

TABLE IV

Results of the ROC curve

Species	ROC curve results
Anopheles forattinii	0.85
Anopheles mattogrossensis	0.81
Anopheles medialis	0.91
Anopheles peryassui	0.78
Kerteszia bellatrix	0.97
Kerteszia cruzii	0.98
Kerteszia homunculus	0.86
Kerteszia lepidota	0.86
Kerteszia neivai	0.89
Nyssorhynchus albitarsis G	0.81
Nyssorhynchus albitarsis H	0.80
Nyssorhynchus aquasalis	0.77
Nyssorhynchus benarrochi B	0.85
Nyssorhynchus braziliensis	0.79
Nyssorhynchus darlingi	0.70
Nyssorhynchus deaneorum	0.88
Nyssorhynchus dunhami	0.87
Nyssorhynchus goeldii	0.88
Nyssorhynchus janconnae	0.91
Nyssorhynchus marajoara	0.83
Nyssorhynchus nuneztovari s.l.	0.81
Nyssorhynchus oswaldoi A	0.85
Nyssorhynchus oswaldoi B	0.89
Nyssorhynchus rangeli	0.71
Nyssorhynchus rondoniensis	0.83
Nyssorhynchus strodei	0.90
Nyssorhynchus tadei	0.88
Nyssorhynchus triannulatus	0.81

After selecting the four most significant variables for each species separate, a MaxEnt model[1] was employed to estimate the potential distribution of all Anophelinae vectors species in Brazil. The logistic model implemented in MaxEnt yielded AUC scores (Table IV), indicating high predictive accuracy.[5]

REFERENCES

1. Phillips SJ, Dudík M, Schapire RE. Maxent software for modeling species niches and distributions (Version 3.4.1). Available from: http://biodiversityinformatics.amnh.org/open_source/maxent/. [Accessed on 2024-10-19].
2. WorldClim. Global Climate Data database. Historical climate data and future climate data. Available from: https://www.worldclim.org/data/ worldclim21.html.
3. Ortega-Huerta MA, Townsend PA. Modeling ecological niches and predicting geographic distributions: a test of six presence-only methods. Rev Mex Biodiv. 2008; 79(1): 205-16.
4. Magurran AE. Measuring biological diversity. Oxford: Blackwell Publishing; 2004. 256 pp.
5. Li X, Wang Y. Applying various algorithms for species distribution modelling. Integr Zool. 2013; 8(2): 124-35. doi: 10.1111/1749-4877.12000.
